# The expression pattern of butyric acid transporter in the large intestine with growth and development of suckling lambs

**DOI:** 10.5713/ab.24.0490

**Published:** 2025-01-24

**Authors:** Jian Ma, Tao Li, Lu Lin, Chunmei Du, Chen Wei, Fuquan Yin, Xuemei Sun, Gang Lyu, Shangquan Gan

**Affiliations:** 1 College of Coastal Agricultural Sciences, Guangdong Ocean University, Zhanjiang, China; 2 Xinjiang Taikun Group Co. Ltd., Changji, China

**Keywords:** Butyric Acid Transporter, Expression Pattern, Large Intestine, Microbial Count, Suckling Lamb

## Abstract

**Objective:**

The objective of this research was to explore the changes of morphological parameters, short chain fatty acid contents and butyric acid transporter expression levels in the large intestine with growth and development of sucking lambs.

**Methods:**

A total of 48 newborn male Hu sheep lambs (body weight = 2.94±0.22 kg) were selected in this experiment. At 0, 7, 14, 28 and 42 days of ages, 6 lambs were slaughtered to collect cecal and colonic samples to analyze morphological parameters, short chain fatty acid contents and butyric acid transporter mRNA expression.

**Results:**

The organ index of the cecum in d 0, 28 and 42 groups was higher (p<0.05) than that in d 7 and 14 groups. Compared with other age groups, the organ index of the colon was significantly increased (p<0.05) in d 42 group. After 7 days of age, the contents of acetic, propionic and butyric acids in the cecum and colon were significantly increased (p<0.05) as age increased. A similar trend of mRNA expressions of butyric acid transporters, including monocarboxylate transporter, sodium-coupled monocarboxylate transporter, sodium-hydrogen exchanger, down regulated in adenoma, putative anion transporter-1 and anion exchanger-2, was found. In the cecum, the *Escherichia coli* count in d 14 group was higher (p<0.05) than that in other age groups, whereas an opposite tendency was found in *Lactobacillus* count between d 14 and other groups. Additionally, in the cecum and colon, the relative expression of claudin-1 in d 14 group was lower (p<0.05) than in d 42 group.

**Conclusion:**

Overall, the current results indicate that the expression levels of butyric acid transporters in the cecum and colon of lambs are significantly influenced by days of age.

## INTRODUCTION

With the improvement of living standards, the consumption of meat, especially mutton and beef, has increased gradually. Mutton and beef are important food sources for humans since they can provide high quality nutrients including protein, fat, minerals and vitamins [1]. The increased demand for mutton and beef has greatly promoted the development of the ruminant breeding industry. In some regions, because of limited resources of available land and immediate needs of grassland ecological protection, the large-scale intensive farming has gradually taken the place of grazing rearing in ruminants’ production. However, the moving space and feed type are changed under intensive feeding, which can have negative effects on ruminants. The increased incidence of mortality and morbidity and reduction of growth rate and immunity in young ruminants decrease the productivity of mutton sheep and beef cattle industries [2,3]. Young stage is an important period that can influence future growth and production performance of adult ruminants. Due to the immature immune system and gastrointestinal tracts, young ruminants are susceptible to pathogenic bacteria that can induce diarrhea and other diseases [4]. Thus, promoting the healthy growth of young animals is of great significance in modern intensive breeding of ruminants.

Accelerating the healthy development of gastrointestinal tracts during early age by utilizing nutritional strategy is an effective method to reduce the occurrence rate of growth retardation, diarrhea and mortality caused by intestinal problems in ruminants [5]. As important fermentation metabolites produced by gastrointestinal microbiota, short chain fatty acid (SCFA) plays an essential role in improving gut health [6]. Acetic, propionic and butyric acids are the most abundant SCFAs, and butyric acid has attracted research attention since it can maintain gut homeostasis [7]. Research has verified that butyric acid or its derivatives supplementation can effectively promote gastrointestinal development by alleviating inflammatory responses, improving intestinal morphology and enhancing mucosal immunity and barrier function [5,6,8]. In ruminants, the rumen is critical to fiber digestion due to the abundance of a large number of microorganisms. In addition, the small intestine is mainly responsible for nutrients digestion and absorption [5,6]. Thus, the rumen and small intestine have received particular attention in ruminants’ study. In fact, the large intestine is also inhabited by a great deal of microorganisms and can degrade fiber and regulate energy metabolism [9]. The SCFA, especially butyric acid, is the main energy source of colonic cells, and plays an essential role in maintaining intestinal health [10]. The butyric acid absorbed by intestinal epithelium has anti-inflammatory and anti-bacteria functions and can also promote the growth and differentiation of colon epithelial cells [11]. At present, most studies of butyric acid and its derivatives in ruminants focus on the rumen and small intestine and pay little attention to the large intestine. Nevertheless, the relationship between butyric acid and large intestine in suckling ruminants is worthy of further study.

Butyric acid is absorbed by gut epithelial cells through a specific carrier-mediated transport system to produce acetyl coenzyme A through oxidation, then producing adenosine triphosphate to provide energy for the gut [12]. During the utilization process of butyric acid, the oxygen is consumed by epithelial cells, which contributes to maintain the anaerobic environment in the gut, and thus prevents the colonization of pathogens such as *Salmonella* and *Escherichia coli* [13]. On the other hand, butyric acid can also enhance the intestinal barrier function by regulating the expression of tight junction proteins, which is conducive to promoting intestinal development [12]. The transporters including monocarboxylate transporter (MCT), sodium-coupled monocarboxylate transporter (SMCT) and sodium-hydrogen exchanger (NHE) are important mediators in the process of butyric acid utilization by intestinal epithelial cells [12,14]. In addition, the dissociated butyric acid is exchanged with HCO_3_^−^ mainly through transporter-mediated butyric acid anion, which consist of down regulated in adenoma (DRA), putative anion transporter-1 (PAT1) and anion exchanger-2 (AE2) [10,12]. Although these transporters can transport other SCFAs, the carriers also have important roles in transporting butyric acid in the intestinal epithelium. Previous studies in ruminants found that the supplementation of SCFA or high-concentrate feeding can regulate the gene expressions of these transporters mentioned earlier in the jejunal and ruminal epithelium [15,16]. In dairy calves, the MCT in the ruminal epithelium begin to express after birth, but the expression is very low [17]. However, the information regarding butyric acid transporters in the large intestine of ruminants is limited. As an important SCFAs, butyric acid can be effectively transported in the intestinal epithelium by the transporters mentioned above. Therefore, in the current study, the MCT, SMCT, NHE, DRA, PAT1 and AE2 were selected as research objectives, and the suckling lambs were used as experimental animals to investigate the expression pattern of butyric acid transporters in the large intestine with growth and development.

## MATERIALS AND METHODS

### Animal ethics statement

In this experiment, all procedures were authorized by the Institutional Animal Care and Use Committee of Guangdong Ocean University (Zhanjiang, Guangdong, China; Approval Code: SYXK-2023-032).

### Experimental design and feeding management

In the current study, a total of 48 newborn male Hu sheep lambs with similar body weight (BW; 2.94±0.22 kg) were selected. At 0, 7, 14, 28 and 42 days of age, 6 lambs of each age were randomly selected and slaughtered. In total, 30 lambs were used in this study. The lambs slaughtered at 0 days of age were not fed colostrum, and the other lambs were given colostrum three times within 18 h after birth (300 mL for the first feeding and 150 mL for the second and third). In this experiment, the colostrum was collected from ewes after parturition by artificial massage of the udder. After collection, the colostrum was placed in an airtight container and stored at a freezer. Before feeding colostrum, the colostrum was thawed in warm water at 36°C. Subsequently, the colostrum was fed to lambs by using a feeding-bottle to ensure the same intake of colostrum. Next, the lambs were fed ewe’s milk three times daily by using bottles. After 35 d, the ewe’s milk was provided twice a day, and then changed gradually to once until weaning at 42 d. The feeding amount of ewe’s milk was 15% of their BW.

All the lambs were separated from their dams immediately after birth. After 7 d of age, all the lambs were provided with alfalfa hay and starter feed for *ad libitum* intake. The composition and nutritional levels of starter are shown in Supplement 1. All lambs were marked with ear tags, and the lambs were reared together in a single hutch (8 m×8 m), which was placed on rice straw that was renewed every three days. Experimental animals had free access to clean water. The temperature of ewe’s milk and water provided for lambs was ranged from 32°C to 36°C.

### Samples collection and analysis

For each age group, 6 lambs were used for samples analysis. After being fasted 12 h, the animals were slaughtered by captive bolt stunning and exsanguinated humanely. The process of slaughter was followed by the National Standard Operating Procedures (GB/T 43562-2023, sheep slaughtering, China). After slaughter, the abdominal cavity of lambs was opened. Then, the cecum and colon were separated with suture line to avoid reflux of contents. The digesta samples from the cecum and colon were collected and put into 10 mL centrifuge tubes at −80°C. Next, digesta samples were used to analyze the SCFA contents including acetic, propionic and butyric acids with gas chromatography (TRACE 1310; Thermo Fisher Scientific, Waltham, MA, USA). In addition, the digesta samples were utilized to determine the microbial composition including *Escherichia coli* and *Lactobacillus* by the method described below. After removing digesta, the cecum and colon were rinsed with ice-clod phosphate-buffered saline, and the weight was recorded. The organ index was calculated by dividing cecal and colonic weights by empty BW.

Subsequently, in the cecum and colon, 2 cm×2 cm tissue samples from mid-portion were collected respectively. The tissue samples were put in 4% paraformaldehyde overnight. Then, tissues were dehydrated and embedded in paraffin and cut into 3 sections of 5 μm for morphological analysis by staining with Hematoxylin and Eosin following the procedures of our previous study [18]. The morphological indexes of the cecum and colon included mucosal and muscular thickness. Additionally, the epithelial tissue (approximately 2 g) of cecum and colon from mid-portion was collected by sterile operating scissors. The samples obtained were washed with phosphate-buffered saline, chopped and placed into 1.5 mL centrifuge tubes. Next, the tubes were stored at −80°C freezer for measurement of mRNA expression of butyric acid transporters and tight junction proteins using quantitative real-time polymerase chain reaction (PCR).

### Microbial composition analysis of digesta samples

The number of *Lactobacillus* and *Escherichia coli* in the digesta samples of lambs was measured using plate count method following the procedures of previous research [19]. Briefly, 1 g of digesta samples from each lamb was evenly mixed with 9 mL sterile water. Subsequently, the mixed liquid were serially diluted to count the *Lactobacillus* and *Escherichia coli*. The small protrusions forming on the De Man-Rogosa-Sharpe plate with slightly white wet and neat edges were regarded as the *Lactobacillus*. In addition, small colonies that formed on the eosin methylene blue plate with purplish black and metallic luster were *Escherichia coli*.

### Quantitative real-time polymerase chain reaction for butyric acid transporters and tight junction proteins

The mRNA expressions of MCT1, SMCT1, NHE1, NHE2, NHE3, DRA, AE2, PAT1, claudin-1, claudin-4, zonula occludens-1 (ZO-1) and occludin in the cecal and colonic tissues were analyzed using real-time PCR technology. Firstly, the tissues samples were used to extract total RNA. Next, the cDNA was reversely transcribed from total RNA by utilizing the cDNA Synthesis kit (Sangon Biotechnology, Shanghai, China) according to the instructions. Quantitative real-time PCR was carried out by the SYBR Green kit (Sangon Biotechnology) and CFX96 Touch PCR System (Bio-Rad Inc., Hercules, CA, USA). The reaction system of PCR is presented in Supplement 2. All the samples were conducted in triplicate. Amplification primers of targeted genes were designed by primer 5.0 software and synthesized from Sangon Biotechnology. The information on amplification primers is shown in Supplement 3. In this experiment, β-actin was used as the housekeeping gene according to the previous study in lambs [2], and the expression levels were obtained by the 2^−ΔΔCt^ method.

### Statistical analysis

Data were based on each lamb as the experimental unit, and the normality of data was assessed by Shapiro-Wilk test of SPSS statistical software (version 22.0). Then, the data were analyzed with one-way ANOVA procedure of the SPSS software. Orthogonal polynomial contrasts were completed to evaluate the linear and quadratic effects according to increase in the ages of lambs. The Duncan test was utilized to determine the differences among different ages. The results of multiple comparisons were corrected by Bonferroni test among the five age groups. After correction, p-values of <0.05 for the multiple comparisons were considered significant. Additionally, correlation analysis (Spearman) between intestinal morphology, tight junction protein expressions and microbial count and butyric acid transporter expressions was carried out using GraphPad Prism software (version 9.0). A p-value lower than 0.05 and the absolute value of the correlation coefficient greater than 0.6 were deemed to be significant correlation.

## RESULTS

### Weight of the cecum and colon and organ index

As shown in Table 1, in the numerical value, the weight of the cecum and colon gradually increased. The cecal weight at 42 days of age was 5.19, 4.22, 3.56 and 1.74 times heavier compared to that at 0, 7, 14 and 28 days of age respectively (p<0.05). In the colon, the weight between 7 and 14 days of age were not significantly different (p>0.05). However, the colonic weight of lambs at d 7 and 14 was higher (p<0.05) than that at d 0, and lower than (p<0.05) that at d 28 and 42. After d 14, the weight of cecum and colon increased rapidly with significant differences (p<0.05). In addition, the organ index of cecum at d 0, 28 and 42 was higher (p<0.05) than that at d 7 and 14. Compared with other age groups, the colonic organ index was significantly increased (p<0.05) in d 42 group.

### Morphology of the cecum and colon

**Figure 1:**
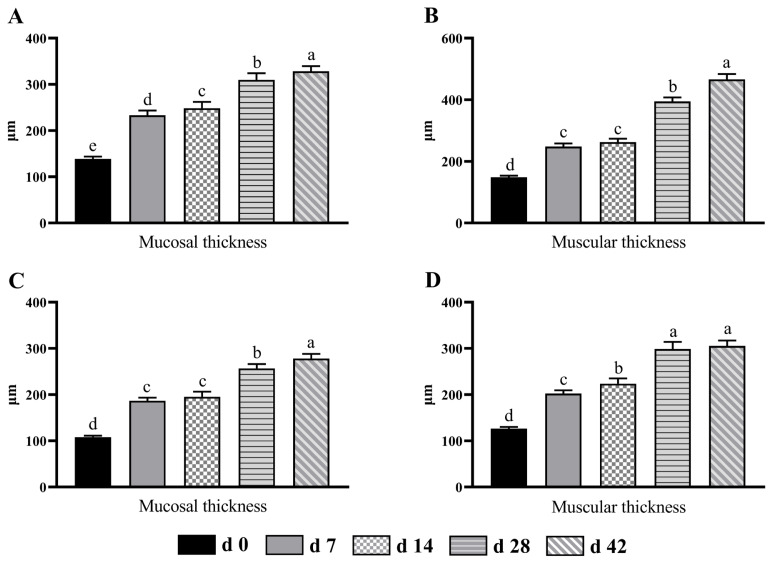
The changes of mucosal thickness and muscular thickness in the cecum (A and B) and colon (C and D) from birth to 42 days of age in sucking lambs. ^a–e^ Columns with different small letters differ significantly (p<0.05).

In the cecum, the mucosal thickness was significantly increased (p<0.05) as the age increased (Figure 1A). The muscular thickness of the cecum in d 42 and 28 groups was significantly higher (p<0.05) than that in other age groups (Figure 1B). There was no significant difference (p>0.05) in the cecal muscular thickness of lambs between d 7 and 14 groups, but it was significantly higher (p<0.05) than that of d 0 group. The variation of the colonic mucosal thickness with individual development of lambs was consistent with that of the cecal muscular thickness (Figure 1C). Compared with other age groups, the muscular layer in the colon of d 28 and 42 groups was significantly increased (p<0.05) (Figure 1D). Moreover, the muscular thickness of sucking lambs showed significant difference (p<0.05) from 0 to 14 days of age.

### Short chain fatty acid content of the cecum and colon

Notably, the concentrations of acetic, propionic and butyric acids in the cecum did not show obvious difference (p>0.05) between 0 and 7 days of age (Table 2). However, after 7 days of age, the contents of acetic, propionic and butyric acids increased gradually with increasing age, and the contents were significantly different (p<0.05) among different age groups. A similar trend of SCFA contents was found in the colon.

### Microbial count of the cecum and colon

**Figure 2:**
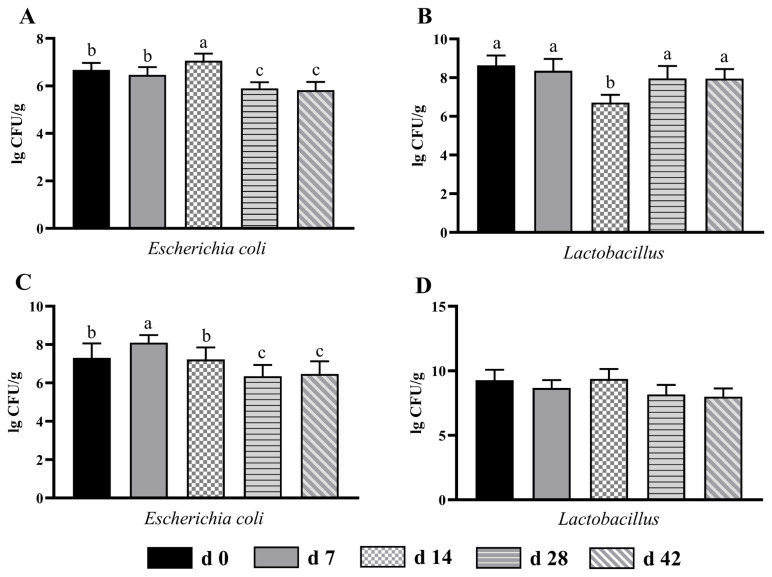
The changes of *Escherichia coli* and *Lactobacillus* counts in the cecum (A and B) and colon (C and D) from birth to 42 days of age in sucking lambs. ^a–c^ Columns with different small letters differ significantly (p<0.05).

As illustrated in Figure 2A, in the cecum, the number of *Escherichia coli* in d 14 group was higher (p<0.05) than that in other age groups, whereas an opposite tendency was found in *Lactobacillus* count between d 14 and other groups (Figure 2B). Additionally, compared with d 0 and 7 groups, the *Escherichia coli* count was significantly reduced (p<0.05) in d 28 and 42 groups. No obvious difference (p>0.05) of *Lactobacillus* count was observed among these groups. In the colon, the *Escherichia coli* count in d 28 and 42 groups was decreased (p<0.05) by 21.48% and 20.12% as compared with d 7 group (Figure 2C). Besides, the *Escherichia coli* count of d 28 and 42 groups was lower (p<0.05) than that of d 0 and 14 groups. However, the number of *Lactobacillus* was similar (p>0.05) among all age groups (Figure 2D).

### Tight junction protein expression of the cecum and colon

**Figure 3:**
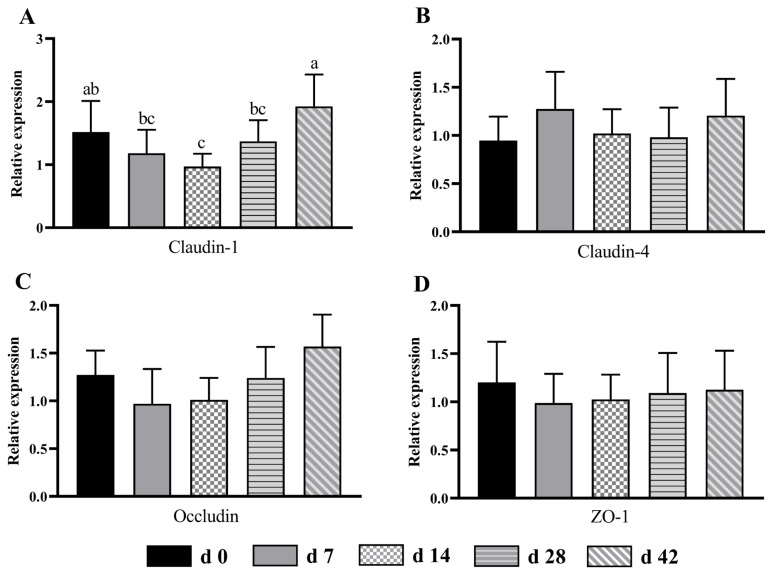
The mRNA expression pattern of tight junction protein in the cecum from birth to 42 days of age in sucking lambs. (A) Claudin-1, (B) claudin-4, (C) occludin, (D) ZO-1. ^a−c^ Columns with different small letters differ significantly (p<0.05). ZO-1, zonula occludens-1.

In the cecum, the relative expression of claudin-1 in d 14 group was lower (p<0.05) than in d 0 and 42 groups (Figure 3A). In addition, compared with d 7 and 28 groups, the claudin-1 expression in d 42 group increased (p<0.05) by 63.56% and 40.88%, respectively. There was no obvious difference (p>0.05) between claudin-4 (Figure 3B), occludin (Figure 3C) and ZO-1 (Figure 3D) mRNA expressions among all age groups.

**Figure 4:**
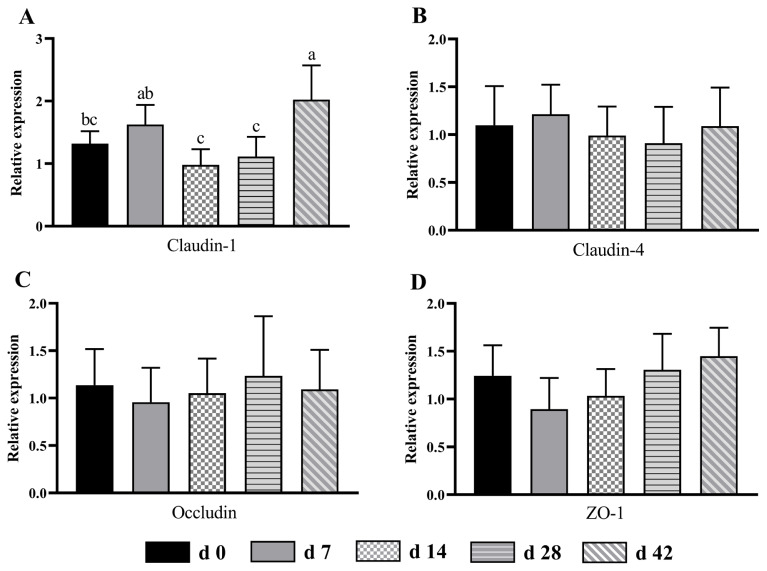
The mRNA expression pattern of tight junction protein in the colon from birth to 42 days of age in sucking lambs. (A) Claudin-1, (B) claudin-4, (C) occludin, (D) ZO-1. ^a−c^ Columns with different small letters differ significantly (p<0.05). ZO-1, zonula occludens-1.

In the colon, the claudin-1 expression of d 42 group was higher (p<0.05) than that of d 0, 14 and 28 groups (Figure 4A). Moreover, compared with d 14 and 28 groups, the mRNA expression of claudin-1 in d 7 group was up-regulated (p< 0.05) by 66.33% and 46.85%, respectively. The expression of claudin-4 (Figure 4B), occludin (Figure 4C) and ZO-1 (Figure 4D) did not show significant difference (p>0.05) among all age groups.

### Butyric acid transporter expression of the cecum and colon

**Figure 5:**
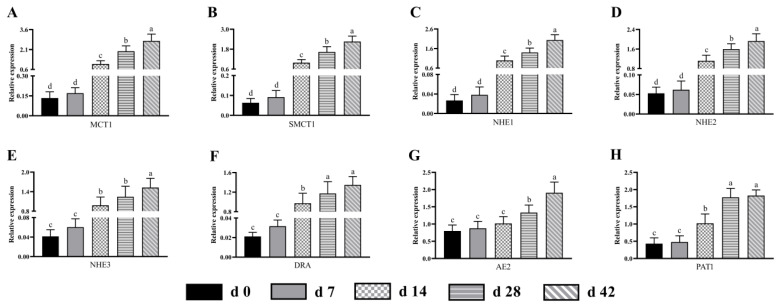
The mRNA expression pattern of butyric acid transporter in the cecum from birth to 42 days of age in sucking lambs. (A) MCT1, (B) SMCT1, (C) NHE1, (D) NHE2, (E) NHE3, (F) DRA, (G) AE2, (H) PAT1. ^a−d^ Columns with different small letters differ significantly (p<0.05). MCT1, monocarboxylate transporter-1; SMCT1, sodium-coupled monocarboxylate transporter-1; NHE1, sodium-hydrogen exchanger-1; NHE2, sodium-hydrogen exchanger-2; NHE3, sodium-hydrogen exchanger-3; DRA, down regulated in adenoma; AE2, anion exchanger-2; PAT1, putative anion transporter-1.

As shown in Figure 5, the mRNA expression levels of butyric acid transporters including MCT1, SMCT1, NHE1, NHE2, NHE3, DRA, AE2 and PAT1 in the cecum were relatively lower before 7 days of age, and the differences were not significant (p>0.05). As the lambs grew order, the expression of these transporters mentioned earlier increased gradually, and the expression levels of MCT1, SMCT1, NHE1, NHE2 and AE2 showed significant changes (p<0.05) among all groups. Apart from PAT1, the expression of other transporters displayed highest value at 42 days of age.

**Figure 6:**
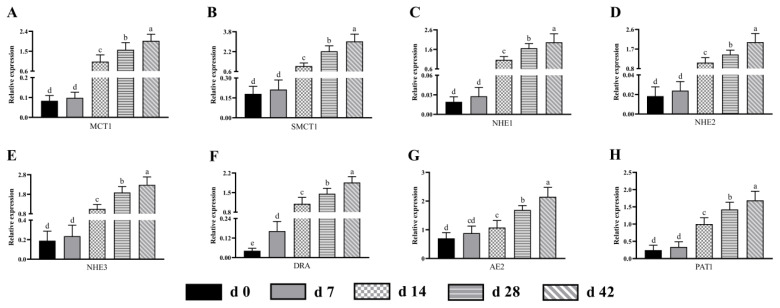
The mRNA expression pattern of butyric acid transporter in the colon from birth to 42 days of age in sucking lambs. (A) MCT1, (B) SMCT1, (C) NHE1, (D) NHE2, (E) NHE3, (F) DRA, (G) AE2, (H) PAT1. ^a−e^ Columns with different small letters differ significantly (p<0.05). MCT1, monocarboxylate transporter-1; SMCT1, sodium-coupled monocarboxylate transporter-1; NHE1, sodium-hydrogen exchanger-1; NHE2, sodium-hydrogen exchanger-2; NHE3, sodium-hydrogen exchanger-3; DRA, down regulated in adenoma; AE2, anion exchanger-2; PAT1, putative anion transporter-1.

Likewise, after 7 days of age, the expression levels of MCT1, SMCT1, NHE1, NHE2, NHE3, DRA, AE2 and PAT1 in the colon of sucking lambs was significantly up-regulated (p<0.05) as age increasing (Figure 6). With exception of DRA, the mRNA expression of other transporters was similar (p>0.05) between d 0 and 7 age groups.

### Association between butyric acid transporter expression and intestinal development, microbial count and tight junction protein expression

**Figure 7:**
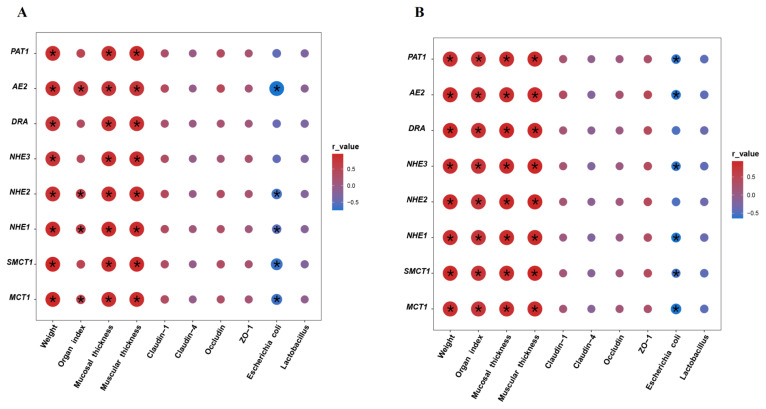
Correlation analysis between butyric acid transporter expression and intestinal development, microbial count and tight junction protein expression in the cecum (A) and colon (B). The circle in red color with asterisk (*) indicates a positive correlation (r>0.6 and p<0.05), and the circle in blue color with asterisk (*) indicates a negative correlation (r<−0.6 and p<0.05). The circle with larger size and darker color represents a higher correlation. PAT1, putative anion transporter-1; AE2, anion exchanger-2; DRA, down regulated in adenoma; NHE3, sodium-hydrogen exchanger-3; NHE2, sodium-hydrogen exchanger-2; NHE1, sodium-hydrogen exchanger-1; SMCT1, sodium-coupled monocarboxylate transporter-1; MCT1, monocarboxylate transporter-1; ZO-1, zonula occludens-1.

Correlation analysis revealed that the butyric acid transporters, including MCT1, SMCT1, NHE1, NHE2, NHE3, DRA, AE2 and PAT1, were positively correlated with the cecal weight, mucosal thickness and muscular thickness (r ranged from 0.759 to 0.953, p<0.05) (Figure 7A). In addition, the MCT1, NHE1, NHE2 and AE2 expression levels had positive correlations with organ index of the cecum (r ranged from 0.603 to 0.718, p<0.05). Nevertheless, an opposite relationship was found between *Escherichia coli* count and MCT1, SMCT1, NHE1, NHE2 and AE2 expressions (r ranged from −0.737 to −0.634, p<0.05). No obvious correlation between tight junction protein and butyric acid transporters was observed (the absolute value of r<0.6 or p>0.05). The same relationship was found between *Lactobacillus* count and butyric acid transporters expressions (the absolute value of r<0.6 or p>0.05).

In the colon (Figure 7B), the mRNA expressions of all butyric acid transporters showed positive correlations with the colonic weight, organ index, mucosal thickness and muscular thickness (r ranged from 0.747 to 0.935, p<0.05). On the contrary, the MCT1, SMCT1, NHE1, NHE3, AE2 and PAT1 were negatively correlated with *Escherichia coli* (r ranged from −0.641 to −0.601, p<0.05). Moreover, there was no obvious relationship between butyric acid transporters and tight junction protein and *Lactobacillus*.

## DISCUSSION

In modern ruminant production, the nutrition of young animals has attracted increasing attention. The healthy feeding of young ruminants has long-term impact on the future growth and production performance of adult animals [2,18]. Under intensive feeding, the early weaning of young ruminants is commonly performed to improve production efficiency. Nevertheless, because of the nutritional and physiological changes of young ruminants during the sucking period, the immature gastrointestinal tracts are sensitive to the changes of external environment, leading to growth retardation and diarrhea, which severely affect the growth and health of young ruminants [2,3]. In the ruminants’ industry, the gastrointestinal dysfunction and low immunity of young ruminants result in increased morbidity and mortality, leading to enormous economic loss [20]. Improving gastrointestinal development is an effective strategy to solve this problem [5]. However, due to the traditional view that the rumen and small intestine are the main digestive organ of ruminants, most studies focused on promoting ruminal and small intestinal development [21,22]. In fact, the large intestine plays an important role in nutrients digestion, energy metabolism and immunity of young ruminants at sucking period [23].

Promoting the large intestinal development of ruminants during the sucking period may be an effective method to improve intestinal health and reduce incidence rate. As one of a key SCFA in the gut, butyric acid is an important energy source for intestine and undertakes a significant role in maintaining healthy intestinal development after being transported by intestinal epithelium [24]. However, up to now, the information of the relationship between butyric acid and large intestine remains scarce. Therefore, in the current study, we used lambs as experimental animals to investigate the changes of butyric acid transporter expression in ruminants with individual development during the sucking stage. Firstly, we studied the developmental characteristics of the cecum and colon of sucking lambs at the age from 0 to 42 d. As expected, the cecal and colonic weights were increased gradually with age increasing. However, in the current study, the weight of the cecum and colon was similar between d 7 and 14 age groups. The supplementation of concentrate or roughage can effectively stimulate the gastrointestinal development of young ruminants [25]. Under the conditions of this experiment, the cecal and colonic development were stimulated by feeding of concentrate and high-quality alfalfa hay from the 7th day. The development of the large intestine of lambs significantly accelerated with the increase of age and adaptation to forage supplementation after 14 d of age.

The weight of the digestive tract can only indicate relative degree of its development, and the morphological parameter of epithelium is tightly linked to nutrients absorption and utilization [26]. Large intestinal epithelium has a vital function in absorption and transport of SCFA, inorganic salt and electrolyte. In the large intestine, the increased mucosal and muscular thickness are beneficial for protecting the intestine from invasion by pathogenic bacteria [27]. With increasing age, the mucosal and muscular thicknesses of the cecum and colon in sucking lambs increased, indicating that the lambs had high immunity and absorption ability at d 42. However, the results require the study of more immune parameters to verify this result. On the other hand, the muscular thickness of the cecum and mucosal thickness of the colon between d 7 and 14 did not show significant difference, which suggested that the development rate of large intestine of lambs was relatively lower during the period that was adapting to solid feed. In addition, the organ index is an important index to reflect the development stage and physiological condition of intestine [28]. For the cecum, the organ index of d 7 and 14 age groups was lower than that of other groups. The possible reason was that the development rate of the cecum in sucking lambs was relatively lower due to the changes of feed type from milk to starter feed and roughage. After adaptation to solid feed, the development rate of cecum in lambs became faster.

In ruminants, the SCFA is the main energy source to maintain physiological metabolism and healthy growth, providing most of the energy requirements [6,24,29]. In our study, the SCFA, including acetic, propionic and butyric acids, in the cecum and colon showed a same variation tendency. Before feeding solid feed, the SCFA in the large intestine of sucking lambs did not show obvious difference. During this stage, sheep milk was the only source of feed, and the fermentation substrate was inadequate. Furthermore, the microbial community in the large intestine was still in developmental stage [4]. Thus, the SCFA production from 0 to 7 days of age was very low. Interestingly, the SCFA was detected in the cecum and colon at d 0 age. This finding was in accordance with previous study in fetal lambs [30], which found that the SCFA was detected in the digesta samples of the cecum. The results found by our and previous experiments indicated that SCFA in the neonatal lambs may not be produced by microbial fermentation in the gut but may also come from dams. Among SCFA, butyric acid is a critical product to accelerate epithelial tissue development of intestine [7]. Previous studies have demonstrated that butyric acid or its derivatives supplementation can effectively promote gastrointestinal development by alleviating inflammatory responses, improving morphology and enhancing barrier function [5,6]. Thus, higher butyric acid concentration in the large intestine might be beneficial for promoting gut development of lambs. After 7 days of age, the butyric acid content in the cecum and colon was significantly increased. Nevertheless, no significant difference in butyric acid concentration was found between d 0 and 7 age groups. Thus, according to the results of our study, 7 days of age may be a suitable time point to regulate butyric acid concentration to promote the development of large intestine.

The microbiota in the intestine not only can utilize nutrients and energy from the feed, but also is a vital barrier again external pathogens, which has an important function in protecting intestinal health and maintaining immunity [4]. Environmental stress as well as immature intestine make sucking lambs vulnerable to the invasion of harmful microorganisms, leading to diarrhea and other diseases [4]. The *Escherichia coli* is the main pathogens that cause diarrhea and threaten the health of lambs. In the cecum, the *Escherichia coli* count of d 14 age group was higher than that of other groups, and in the colon, the d 7 age group had highest count of *Escherichia coli*. The result indicated that the intestinal immunity of sucking lambs was relatively lower during the adaptation period to solid feed. Thus, during this period, feeding management of lambs should be strengthened to attenuate stress caused by change of feed type and reduce the incidence of diarrhea. *Lactobacillus* can generate lactic acid by using carbohydrate in the gut to create an acid environment, which then inhibits the reproduction and colonization of pathogenic bacteria [31]. A previous study has reported that the increased *Lactobacillus* count was positively related to daily weight gain and feed conversion efficiency [19]. Our experiment found that the *Lactobacillus* count in the cecum was lower in d 14 age group, which was matched to *Escherichia coli* result, further suggesting the importance of feeding management during the adaptation to solid feed. After 28 days of age, the number of *Escherichia coli* and *Lactobacillus* tended to be stable. Compared with early stages, the *Escherichia coli* and *Lactobacillus* counts were reduced after supplementation with solid feed. This result was partly attributed to the increase of age of lambs, the quantity of intestinal contents gradually increased, and the number of microorganisms per unit in contents was diluted.

The tight junction between intestinal cells is an important structural basis for intestinal barrier function and plays an important role in regulating the intestinal permeability and maintaining the epithelial cell barrier [32]. In ruminants, many diseases are related to the abnormal expression of tight junction protein, such as growth retardation, diarrhea and intestinal inflammation [33,34]. In the current study, the mRNA expression of claudin-1 in the cecum and colon was lower in d 14 age group than that in d 42 age group, indicating that the lambs in d 42 group had healthier intestines. A previous study in Holstein male calves found that *Escherichia coli* infection could down-regulate the gene expression of tight junction proteins, including claudin-1, occludin and and ZO-1 [35]. Our tight junction protein expression results were in line with microbial count findings, indicating that the lambs were susceptible to pathogenic bacteria, and then damaging the intestinal barrier function. The metabolites of intestinal microbiota including butyric acid is verified to regulate the expression of tight junction protein [5]. Thus, future studies to explore the function of microbial community by metagenomics and metabolites by metabonomics are needed to obtain more information on the role of microbial community in the large intestine of sucking lambs.

As mentioned earlier, butyric acid are quantitatively important substrates in energy metabolism of ruminants [12]. The absorption of butyric acid in the intestine that occurs across epithelium is a complicated process, and acts as a vital energy source for maintaining intestinal health [36]. As a weak monocarboxylic acid, most butyric acid exists in the gastrointestinal tracts as acid ions. Under ionic form, the transportation of butyric acid in the intestinal epithelium is a more complicated process, which affects its diffusion, thus the transmembrane transportation is regarded as the main way of butyric acid absorption [37]. Early study has reported that the transportation of butyric acid in the colon is conducted by a transporter-mediated mechanism [38]. Unfortunately, at present, the expression pattern of butyric acid transporters has not been investigated in the large intestine of ruminants. In this study, as age increased, the expression levels of butyric acid transporters, including MCT1, SMCT1, NHE1, NHE2, NHE3, DRA, AE2 and PAT1, were gradually up-regulated in the cecum and colon of lambs. A previous study compared the mRNA expression of MCT1 in the rumen between adult cows and calves, and results showed that the expression of MCT1 in adult cows was higher than that in calves [17], which was basically in line with our study. In addition, exception of DRA in the colon, the expressions of other transporters in the cecum and colon did not show significant difference between d 0 and 7 age groups, indicating that before supplementation of solid feed, the ability of butyric acid transportation was lower. Our finding verified that despite the expression level being low, the butyric acid transporters in the cecum and colon begin to express after birth. This expression pattern of butyric acid transporters in the large intestine provides a possibility for researchers to regulate its expression through external intervention such as nutritional strategy. According to the results of the current study, the appropriate time point is 7 days of age.

Correlation analysis found that the butyric acid transporters expression levels were positively associated with the cecal and colonic development parameters including intestinal weight, mucosal thickness and muscular thickness, indicating that promoting the butyric acid carriers’ expression may be beneficial for large intestinal development. In addition, the butyric acid transporters expressions were negatively related to *Escherichia coli* count. Previous studies in lambs and calves have revealed that accelerating intestinal development by dietary supplementation with feed additives can effectively reduce the diarrhea rate, thus improving the healthy growth of animals [39,40]. The positive relationship between butyric acid transporters and intestinal development and negative relationship between transporters and *Escherichia coli* indicated that promoting the butyric acid transporters expression may be helpful for enhancing the energy utilization of intestine to reduce morbidity and maintain the growth of sucking lambs. Due to the role of the foregut of animals, the small amount of butyric acid contained in milk has difficulty reaching the large intestine. Therefore, in the following study, we will use an embedding technique to increase the amount of butyric acid reached the large intestine of sucking ruminants and study its influence on the expression of butyric acid transporters in the large intestinal epithelium.

## CONCLUSION

The results from the current study provide evidence that with age increasing, the butyric acid concentration and transporters expression including the MCT1, SMCT1, NHE1, NHE2, NHE3, DRA, AE2 and PAT1 increase gradually in the cecum and colon of sucking lambs. In addition, between 7 to 14 days of age, the claudin-1 expression in the large intestine is reduced, where the *Escherichia coli* count is increased. Correlation analysis results showed that the butyric acid transporter expression has positive relationship with intestinal development and negative relationship with *Escherichia coli* count. Based on the findings of our study, improving the expression levels of butyric acid transporters in the large intestine may be an effective strategy to promote intestinal development of sucking lambs.

## Figures and Tables

**Table 1:** Effects of day of age on the cecal and colonic weights and organ index in lambs

Items	Day of age					SEM	p-value		
	
	0	7	14	28	42		Treatment	Linear	Quadratic
Weight (g)									
Cecum	5.66^d^	6.96^cd^	8.25^c^	16.86^b^	29.38^a^	1.665	<0.001	<0.001	<0.001
Colon	12.27^d^	31.86^c^	34.25^c^	72.78^b^	145.97^a^	8.840	<0.001	<0.001	<0.001
Organ index (%)^1)^									
Cecum	0.192^b^	0.139^c^	0.129^c^	0.187^b^	0.244^a^	0.008	<0.001	<0.001	<0.001
Colon	0.415^e^	0.635^c^	0.537^d^	0.808^b^	1.216^a^	0.052	<0.001	<0.001	<0.001

1) The organ index was calculated by dividing cecal and colonic weights by empty body weight of lambs.

a–e In the same row, values with different superscripts mean significant differences (p<0.05).

SEM, standard error of the mean.

**Table 2:** Effects of day of age on the cecal and colonic short chain fatty acid content in lambs

Items	Day of age					SEM	p-value		
	
	0	7	14	28	42		Treatment	Linear	Quadratic
Cecum (mg/g)									
Acetic acid	4.2×10^−3^^d^	0.021^d^	1.348^c^	2.56^b^	4.23^a^	0.300	<0.001	<0.001	<0.001
Propionic acid	6.25×10^−5^^d^	1.24×10^−4^^d^	0.467^c^	1.26^b^	2.04^a^	0.147	<0.001	<0.001	<0.001
Butyric acid	3.42×10^−4^^d^	8.77×10^−4^^d^	0.275^c^	0.413^b^	0.950^a^	0.065	<0.001	<0.001	<0.001
Colon (mg/g)									
Acetic acid	2.92×10^−4^^d^	1.18×10^−3^^d^	1.01^c^	1.717^b^	2.78^a^	0.197	<0.001	<0.001	<0.001
Propionic acid	8.67×10^−6^^d^	2.63×10^−5^^d^	0.315^c^	0.643^b^	1.16^a^	0.082	<0.001	<0.001	<0.001
Butyric acid	7.23×10^−5^^d^	1.41×10^−4^^d^	0.092^c^	0.273^b^	0.527^a^	0.037	<0.001	<0.001	<0.001

a–d In the same row, values with different superscripts mean significant differences (p<0.05).

SEM, standard error of the mean.

## SUPPLEMENTARY MATERIAL

**Supplement 1.** Composition and nutrient levels of the starter (DM basis)

**Supplement 2.** The reaction system of real-time PCR

**Supplement 3.** Primers used for quantitative real-time PCR of lambs in this study
