# Supplementary material for: The expression pattern of butyric acid transporter in the large intestine with growth and development of suckling lambs
Source: Anim Biosci. 2025 Jan 24;38(5):968–80. doi: 10.5713/ab.24.0490 (PMC12062808; doi:10.5713/ab.24.0490)
Supplement: Supplementary file 1 [file ab-24-0490-Supplementary-1.pdf]

Supplement 1

Composition and nutrient levels of the starter (DM basis).

Ingredients, %	Content	Nutrient levels	Content
Corn	55.80	CP (%)	18.72
Soybean meal	20.20	EE (%)	5.40
Cottonseed mean	18.70	NDF (%)	21.67
NaCl	0.30	ADF (%)	8.11
$NaHCO_3$	0.60	Ca (%)	0.78
$CaHPO_4$	0.40	P (%)	0.41
Premix[1]	4.00	ME[2] (MJ/kg)	17.62

DM, dry matter; CP, crude protein; EE, ether extract; NDF, neutral detergent fiber; ADF, acid detergent fiber; ME, metabolizable energy.

[1] The premix provided following per kilogram of diet: Fe 280 mg, Zn 50 mg, Mn 40 mg, Cu 10 mg, I 0.60 mg, Se 0.40 mg, Co 0.20 mg, VA 8000 IU, VD 900 IU, VE 30 IU.

[2] ME was a calculated value; the other nutritional levels were measured values.
