# Supplementary material for: The expression pattern of butyric acid transporter in the large intestine with growth and development of suckling lambs
Source: Anim Biosci. 2025 Jan 24;38(5):968–80. doi: 10.5713/ab.24.0490 (PMC12062808; doi:10.5713/ab.24.0490)
Supplement: Supplementary file 2 [file ab-24-0490-Supplementary-2.pdf]

Supplement 2

The reaction system of real-time PCR.

Reagents	Volume, μL
2×Real PCR SYBR Green	10.0
Forward primer (10 μM)	1.0
Reverse primer (10 μM)	0.4
Template cDNA	0.4
ddH_2O	8.2
Total	20.0
