# Supplementary material for: The expression pattern of butyric acid transporter in the large intestine with growth and development of suckling lambs
Source: Anim Biosci. 2025 Jan 24;38(5):968–80. doi: 10.5713/ab.24.0490 (PMC12062808; doi:10.5713/ab.24.0490)
Supplement: Supplementary file 3 [file ab-24-0490-Supplementary-3.pdf]

Supplement 3

Primers used for quantitative real-time PCR of lambs in this study.

Genes	Primer sequence (5'-3')	GenBank ID	Amplicon size, bp
β-actin	F: TCCGTGACATCAAGGAGAAGC<br>R: CCGTGTTGGCGTAGAGGT	NM_001009784.2	266
MCT1	F: GTGGCTTGATTGCTGCTTCC<br>R: GCCAATCATGGTCAAAGCCG	XM_060412888.1	116
SMCT1	F: GTATTGGCATGGCTGCACTG<br>R: TGAGTTGGCAAAGGGAACCA	XM_004006660.5	131
NHE1	F: CACCACTGGAACTGGACGTT<br>R: TGGGGGTCAACTTCACGATG	XM_004005085.5	121
NHE2	F: GATCACGTGAAGACCGGGAT<br>R: TGGGTTGGTTCTCCCGAATC	XM_027963443.1	124
NHE3	F: TCATCATCGTCTTCGGGGAG<br>R: TTCCTTTCACGCAATCCACG	XM_042233997.2	123
DRA	F: CCTACAGGAATCGTGGGCTA<br>R: CTCGGCTGATTCCTCAGGGT	NM_001001389.2	82
AE2	F: GGTGCGGAAGAATGCCAAAG<br>R: CCTGGTTTTTGTCCAGCAGC	XM_046951970.1	146
PAT1	F: GCCTTTCGTGGACTATGGGG<br>R: AGCTGCGTGACGATTAGGAA	XM_023452815.2	123
Claudin-1	F: AATACATTGAGGTCACCGAGTA<br>R: GATTAGGCAAGGAAAGGCAC	NM_001185016.1	191
Claudin-4	F: GCCTTCATCGGCAGCAACAT<br>R: CCAGCAGCGAGTCGTACACCTT	NM_001185017.1	115
Occludin	F: AGTGGTAACTTGGAGACGCTTTC<br>R: CCTCCCGTCGTGTAGTCTGTT	XM_012145891.2	107
ZO-1	F: CATCACGCCAGCATACAA<br>R: GCAGACTTCAGGAGGGTTT	XM_042235171.1	177

MCT1, monocarboxylate transporter-1; SMCT1, sodium-coupled monocarboxylate transporter-1; NHE1, sodium-hydrogen exchanger-1; NHE2, sodium-hydrogen exchanger-2; NHE3, sodium-hydrogen exchanger-3; DRA, down regulated in adenoma; AE2, anion exchanger-2; PAT1, putative anion transporter-1; ZO-1, zonula occludens-1.

F, forward; R, reverse.
